# Discrepancy in applied tidal volumes between t-piece and lung level during delivery room stabilization – a case report

**DOI:** 10.3389/fped.2026.1918821

**Published:** 2026-08-21

**Authors:** Alexia Zanghi, Maria Wall, David G. Tingay, Andreas W. Flemmer, Vincent D. Gaertner

**Affiliations:** 1 Division Neonatology Department of Pediatrics, Dr. von Hauner Children’s Hospital, LMU Medizin, LMU University Hospital, Ludwig-Maximilians-Universität München, Munich, Germany; 2 Neonatal Research, Murdoch Children’s Research Institute, Melbourne, VIC, Australia; 3 Department of Paediatrics, University of Melbourne, Melbourne, VIC, Australia; 4 Member of the German Center for Lung Research (DZL), Comprehensive Pneumology Center Munich, Munich, Germany

**Keywords:** case report, delivery room, electrical impedance tomography, neonatal resuscitation, respiratory function monitor

## Abstract

We present data of a preterm infant at 28 4/7 weeks gestation with inadequate response to non-invasive ventilation after birth who was intubated five minutes after delivery. During non-invasive stabilization, we noted a discrepancy between the displayed tidal volumes on the respiratory function monitor (RFM) and the real-time electrical impedance tomography (EIT) measurements. While expiratory tidal volumes on the RFM were in the recommended target of 4–6 mL/kg, EIT showed only minimal tidal volumes (corresponding to approximately 1–3 mL/kg), which was more consistent with the clinical impression of the neonate. After intubation, tidal volumes shown on RFM and EIT were similar, suggesting that RFM may be unreliable during non-invasive ventilation, possibly due to the large compliance of the nasopharyngeal space. EIT may be a helpful additional clinical tool in selective cases in the delivery room.

## Highlights

### Established facts

- Current recommendation for expiratory tidal volumes for delivery room stabilization of preterm infants is 4–6 mL/kg (1).
- Lower tidal volumes lead to ineffective ventilation, while higher volumes are associated with higher risk of intraventricular haemorrhage (1).
- Respiratory function monitors may be sensitive to various confounding factors (2, 3).
- Electrical impedance tomography (EIT) is a radiation-free, non-invasive method of measuring tidal volume distribution inside the lungs in real-time (4–6).

### Novel insights

- Clinicians may need to interpret RFM-displayed tidal volumes with awareness of their limitations.
- EIT may be considered as a complementing monitoring tool in the delivery room to visualize intrapulmonary volume changes, especially in very preterm infants.

## Introduction

Current recommendation for expiratory tidal volumes for delivery room stabilization of preterm infants is 4–6 mL/kg to allow effective ventilation without causing intraventricular hemorrhage (1). Respiratory function monitors (RFMs) are used for tidal volume targeting but may be sensitive to confounding factors (2, 3). Electrical impedance tomography (EIT) is a non-invasive method of measuring lung ventilation (4–6). Here, we present a preterm infant with normal to high expiratory tidal volumes on the RFM (VT_RFM_) despite limited lung ventilation as indicated by EIT (VT_EIT_, a retrospectively calibrated EIT-derived estimate of V_T_) in the delivery room (DR).

## Case presentation

The patient was included in a prospective observational study (ethics number: 25-0551). Written informed consent was obtained by the parents for publication of this case report.

The resuscitation was video recorded. Respiratory function parameters were recorded at 200 Hz using the NewLifeBox (Advanced Life Diagnostics, Weener, Germany). In this case, the Sophie A pneumotachograph by Fritz Stephan GmbH was used in the delivery room which compensates for different gas compositions. An EIT-belt was fastened around the infants' chest, and EIT data were continuously recorded at 50 Hz using the LuMon device (SenTec AG, Landquart, Switzerland). This manuscript follows the CARE case report guidelines.

During delivery room stabilization, an additional observer monitored the EIT display and communicated qualitative information regarding intrapulmonary ventilation to the treating clinician in real time, whereas the EIT-derived VT values in mL/kg were calculated retrospectively using calibration data obtained after intubation.

RFM and EIT data were extracted in 30-s sequences every 30 s if there were enough artefact-free breaths discernible in both devices. Matching was not performed for each single breath but rather for these entire 30 s sequences. EIT data was analyzed as previously described (4, 5, 7). VT_EIT_ is usually measured in arbitrary units. Thus, we used a period of stable leak-free ventilation several minutes after intubation (between 07:09 and 07:39 min after birth) and compared VT_EIT_ to VT_RFM_ in 24 temporally matching breaths to establish a conversion factor. The conversion factor was calculated as the ratio of the mean EIT signal to the mean RFM derived tidal volumes across these breaths. This is generally accepted to confirm the absolute magnitude of EIT-derived VT and represents absolute tidal volumes as accurately as possible. This factor was then used to retrospectively express the EIT signal in mL/kg as an RFM-referenced, absolute-scale estimate of intrapulmonary tidal volume for all extracted timepoints and will be referred to as VT_EIT_. Statistical analysis was performed using R studio (Version 2025.09.2 + 418). Median and IQR were calculated for each timepoint.

The infant was delivered via cesarean section with general anesthesia after one dose of antenatal corticosteroids at 28 ^4/7^ weeks gestation due to suspected chorioamnionitis. Birth weight was 1,150 g.

**Figure 1:**
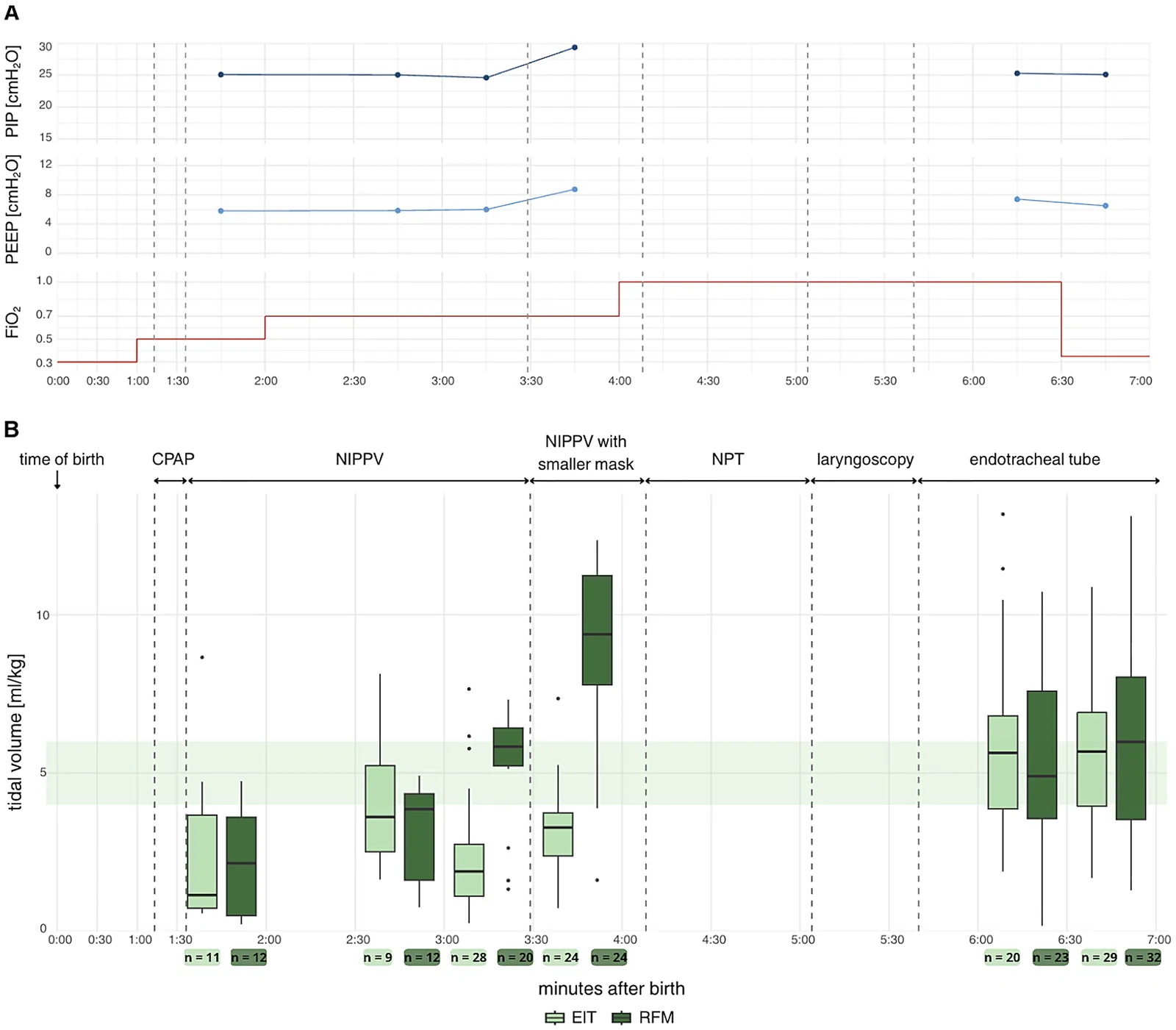
Tidal volumes from RFM and EIT for the first seven minutes after birth. (**A**) set PIP, PEEP and FiO_2_ across time; (**B**) comparison of tidal volumes and course of clinical management. The target range of tidal volume is highlighted in green. Of note, leak was minimal during all measurements. No EIT data was available until starting NIPPV, and no flow data was available during NPT ventilation and laryngoscopy. The time period when the conversion factor was calculated was several minutes after intubation and is thus not part of the figure. *n* indicates the number of breaths included in the analysis derived from EIT and RFM measurements. CPAP, continuous positive airway pressure; NIPPV, non-invasive positive pressure ventilation; NPT, nasopharyngeal tube; EIT, electrical impedance tomography; RFM, respiratory function monitor; PIP, positive inspiratory pressure; PEEP, positive end-expiratory pressure.

After 60 s delayed cord clamping the infant was brought to the resuscitaire. It was started on continuous positive airway pressure support (CPAP) using a T-piece ventilation device (Perivent®). The pulse oximeter did not show any values until 5 min after birth. The infant developed secondary apnea and non-invasive positive pressure ventilation was initiated. Based on clinical impression (blue skin color, low muscle tone), FiO_2_ was increased stepwise to 1.0. Also, mask was changed to a smaller size, the head was repositioned and the positive inspiratory pressure was raised from 25 to 29 cmH_2_O before inserting a nasopharyngeal tube (NPT; Figure 1A) (8). The infant was intubated one minute later due to persistently inadequate ventilation. After intubation, SpO_2_ increased and FiO_2_ and PIP could be reduced.

While EIT and RFM initially both showed low tidal volumes, VT_RFM_ was within target range after mask repositioning [median 5.8 mL/kg (IQR 5.2 to 6.4)] and even increased above target range after mask change (Figure 1B). Within the corresponding 30-s interval, VT_EIT_ was below target [2.4 mL/kg (IQR 1.4 to 3.7)], consistent with the poor clinical impression of the neonate. After intubation, this discrepancy was resolved with similar tidal volumes in both devices [VT_RFM_ 6.0 mL/kg (IQR 3.5 to 7.9) vs. VT_EIT_ 5.7 mL/kg (IQR 4.0 to 6.9), Figure 1B]. During stabilization, leak was consistently minimal and no obstruction was present (defined as V_T_ < 2 mL/kg when leak <30%). During NPT ventilation and laryngoscopy, there was no RFM data available. Surfactant was given in the DR, extubation followed <24 h later and treatment on the NICU was uncomplicated; respiratory support was terminated on day 38 and cranial ultrasound showed no abnormalities.

## Discussion and conclusion

We present a case of a preterm neonate with adequate tidal volumes measured via flow sensor at the t-piece but inadequate VT at lung level using a retrospectively calibrated EIT-derived estimate of VT. This indicates that using an RFM during DR stabilization may not always be sufficient and EIT may provide additional important information on lung aeration and ventilation.

After initial mask and head repositioning, VT_RFM_ were constantly in or above target range (1). but VT_EIT_ were minimal until intubation. The reliability of tidal volumes displayed on RFMs has been questioned previously (2, 3). First, mask dead space and high oropharyngeal compliance may contribute to inflations only distending mask and oropharynx, leading to measurable (or even adequate) tidal volumes at facemask level despite inadequate intrapulmonary tidal volumes. This also explains why EIT-derived VT were consistently smaller during non-invasive ventilation than after intubation. Second, flow sensors are calibrated using room air and the higher viscosity of increased oxygen may cause an overestimation of tidal volumes (9). However, the used flow sensor compensates for different gas compositions and we did not have data available when FiO_2_ was 1.0, making it unlikely that this is the major contributing factor. Theoretically, condensation inside the respiratory system may also falsely suggest high V_T_ but here, no humidified gas was used (2). Still, a visible RFM may be helpful to guide ventilation in certain cases if used and interpreted adequately.

In our case, the EIT monitor showed only little to no ventilation, consistent with the poor clinical impression of the neonate. EIT measures impedance changes in real time at lung level, thus allowing detection of poor breathing patterns (4–6) which helped determine that the lung was not ventilated in this infant. Still, there are some limitations: 1) EIT does not measure absolute VT which limits inference on the magnitude of tidal volumes. Offline calibration with a ventilator was needed to calculate VT_EIT_ and detect RFM inaccuracy. 2) The conversion factor was calculated using breaths of an intubated infant. Due to various reasons (glottal closure, irregular breathing patterns, gastric inflation, high nasopharyngeal compliance, etc) lung volume changes may be smaller during non-invasive compared with invasive ventilation. Indeed, this is in line with our findings where changes at lung level were consistently lower during non-invasive ventilation. This is based on a series of clinical phenomena during different physiological conditions of the neonate. 3) Pairing of EIT- and RFM-derived VT was not performed using an individual breath-by-breath synchronization but rather using time intervals of 30 s. This sequence matching still allowed accurate enough comparison of devices. Within these sequences, the same breaths are included without exact one-to-one matching of all breaths. 4) The measured leak does not take into account the air that inflates the esophagus. Therefore, the actual leak might be slightly higher than displayed. However, this may also serve as part of the explanation for the discrepancy between devices and serves as another indicator that EIT may be beneficial in some situation such as the DR.

This case report indicates that EIT may be beneficial in the DR where ventilation is anticipated to be challenging (7, 10). This adds to the growing evidence of its usefulness on the NICU and in the DR (4). However, whether this translates into improved outcomes needs to be evaluated in randomized controlled studies.

In conclusion, our findings suggest that 1) clinicians may need to interpret RFM-displayed tidal volumes with awareness of their limitations, especially during non-invasive ventilation and 2) EIT may complement RFM and clinical assessment by providing information on the presence and relative magnitude or distribution of intrapulmonary ventilation.

## Data Availability

The raw data supporting the conclusions of this article will be made available by the authors, without undue reservation.
